# InterNet+: A Light Network for Hand Pose Estimation

**DOI:** 10.3390/s21206747

**Published:** 2021-10-11

**Authors:** Yang Liu, Jie Jiang, Jiahao Sun, Xianghan Wang

**Affiliations:** College of Systems Engineering, National University of Defense Technology, Changsha 410073, China; yangliu@nudt.edu.cn (Y.L.); sunjiahao@nudt.edu.cn (J.S.); 18373140341@163.com (X.W.)

**Keywords:** hand pose estimation, neural network, attention mechanism

## Abstract

Hand pose estimation from RGB images has always been a difficult task, owing to the incompleteness of the depth information. Moon et al. improved the accuracy of hand pose estimation by using a new network, InterNet, through their unique design. Still, the network still has potential for improvement. Based on the architecture of MobileNet v3 and MoGA, we redesigned a feature extractor that introduced the latest achievements in the field of computer vision, such as the ACON activation function and the new attention mechanism module, etc. Using these modules effectively with our network, architecture can better extract global features from an RGB image of the hand, leading to a greater performance improvement compared to InterNet and other similar networks.

## 1. Introduction

Considering the continuous development of information techniques, various electronic devices equipped with artificial intelligence systems have been integrated into our lives [1,2], and the demand for human–computer interaction has become increasingly high. As the basic work of hand-based interaction, human hand pose estimation and recognition also have a great research value [3]. There are many methods for hand pose estimation using deep learning. These include methods based on images with depth information [4,5,6,7], methods directly based on RGB images [8,9,10,11], and methods based on binocular or even multi-eye visions [12,13]. Among them, the hand pose estimation method based on single RGB image has attracted more attention because of its simple implementation, smaller hardware requirement, and ease of its promotion.

Using deep learning methods to estimate hand pose based on a RGB image, one of the possible methods is InterNet, which was introduced by [8]. InterNet accurately estimates the posture position of the hand by inputting an annotated RGB image using a deep neural network feature extractor and subsequent heatmap estimation and position-fitting with the fully connected network. As the baseline of the InterHand2.6M dataset proposed by the author, this method improves the performance of hand pose estimation, implements it accurately on the STB (stereo hand pose tracking benchmark) dataset, and solves the problem of interactive hand pose estimation.

Still, as the single structure of the feature extraction network in the original InterNet, it has huge potential for improvement. In the years after the publication of the original InterNet network, tremendous progress has been made in the field of machine learning and deep neural networks [8]. For the purpose of achieving the potential of the original InterNet structure and verify whether the current achievements can be effectively applied to the field of hand pose estimation, as well as improving the performance in multiple datasets, we relied on the recent developments in this field to update the original method and achieved greater improvements on multiple datasets. Therefore, we refer to the resulting network as InterNet+. The scope of the study is summarized as follows:

- We redesigned a feature extractor based on deep neural networks to replace the simpler ResNet-50 [14] backbone architecture in the original network and continue to ensure the overall lightweight of the network. We refer to the architecture design of MobileNet v3 [15] and MoGA [16] network and introduce the inverted residual block, Hsigmoid, and Hswish activation functions [15,17]. The latest coordinate attention mechanism [18] is introduced in the bottleneck structure and part of the latest ACON (Activation or Not, can choose the linear or non-linear structure with self-learning) activation function [19];
- We introduced the multi-spectral attention mechanism named FcaNet [20] to process the obtained feature maps before fully connected network in order to retain more frequent domain information to improve the performance;
- We tried to improve the overall training procedure of the network to obtain more information from the available data.

Subsequently, we will introduce the related methods of hand pose estimation based on deep learning in Section 2, briefly review the original InterNet architecture and data labeling in Section 3, and introduce our modifications in Section 4. Section 5 evaluates these modifications and compares them with the latest methods on public benchmark datasets.

## 2. Related Work

In this section, we will briefly introduce the related work on hand pose estimation based on deep learning. There are several preliminary studies on hand pose estimation. We refer to [2,3] as well as our previous review on hand pose estimation [21] and recent studies for a brief overview.

Considering a previous study, depth map was mainly used for depth information extraction and position information inference for hand pose estimation [2]. DeepPrior [22] used a convolutional neural network (CNN) structure to estimate the joint points’ 3D-position of the hand through a depth map. By introducing prior knowledge about the 3D pose, the author significantly improved the accuracy and reliability of hand pose estimation. The follow-up DeepPrior++ [4] added residual network structure [14], data enhancement processing, and better initial hand localization to improve the network performance. Moon et al. [23] converted the 3D hand and human pose estimation problem from a depth map to a voxel-to-voxel prediction, which used a 3D voxelized grid and estimated the per-voxel likelihood of each key-point. Zhu et al. [24] used the point set method, proposed an adaptive pooling for the network to select features by itself, and proposed an integration strategy that made total use of hand features. Moreover, Rong et al. [7] used Fourier descriptors to recover the depth information of hand gestures from the depth map.

In 2017, Zimmermann et al. [11] used CNN structure for hand pose estimation. This shifted the task gradually to the direction of hand depth information recovery and pose estimation using RGB images. They cleverly designed HandSegNet, PoseNet, and PosePrior networks to complete the three tasks of segmenting the hand region from the image, performing pose estimation and fitting, as well as mapping it to the 3D space to infer its 3D position, respectively. Cai et al. [25] proposed a weakly supervised 3D hand pose regression network, which used depth maps to compensate the absence of entire 3D annotations. Moon et al. [8] proposed InterNet for multi-targets (two or less) and interactive hand pose estimation. The specific implementation method will be introduced in detail in Section 3. Ge et al. [9] designed a sophisticated network using a simplified linear graph convolutional neural network to perform linear regression from the reconstructed 3D hand mesh vertices to complete the task of hand pose estimation. The convolutional neural network is used to extract the global features of the RGB image to synthesize the potential feature vectors, as these features are up-sampled, convolved, and assigned to a finer image. Li et al. [26] used learnable joint groups for hand pose estimation, which can automatically group the hand joints. They used the principle of multi-task learning (MTL) by separating less-related joints into different groups as different tasks and therefore efficiently avoided the negative transfer among less related tasks. X. Chen et al. [27] presented a camera-space mesh recovery (CMR) framework to unify tasks of root-relative hand mesh recovery and hand root recovery. They proposed an aggregation method to collect effective 2D cues and then captured high-level semantic relations through aggregation of joint semantics, which is instructive for root-relative mesh recovery. To alleviate reliance on labeled training data, Y. Chen et al. [28] designed S^2^ HAND, a self-supervised 3D hand reconstruction network, which can jointly estimate pose, shape, texture, and the camera viewpoint.

For hand-object pose estimation, Bardia et al. [29] proposed HOPE-Net, based on the Graph-CNN, to complete hand-object pose estimation tasks. The network used ResNet-10 as an image encoder to predict the initial 2D coordinates of joints and object vertices. The coordinates connected with the image features are used as the features of the input graphics of the three-layer graphics convolution to use the power of the neighboring features to estimate a better 2D pose. Finally, the 2D coordinates predicted in the previous step are passed to the adaptive Graph U-Net to find the 3D coordinates of the hand and the object. Y. Chen et al. [30] presented an end-to-end feature fusion network to recover 3D shapes of hand and object. They used the depth map predicted from the input RGB to enhance the spatial attribute, then proposed a long short-term memory (LSTM) feature fusion module, which effectively enhanced the object feature after comparing multiple feature fusion strategies.

## 3. Original InterNet

In this section, we will briefly review the original InterNet architecture and its related data processing procedure to contrast with our modifications to the network in Section 4. For further details, please refer to [8].

### 3.1. Network Structure

**Figure 1:**
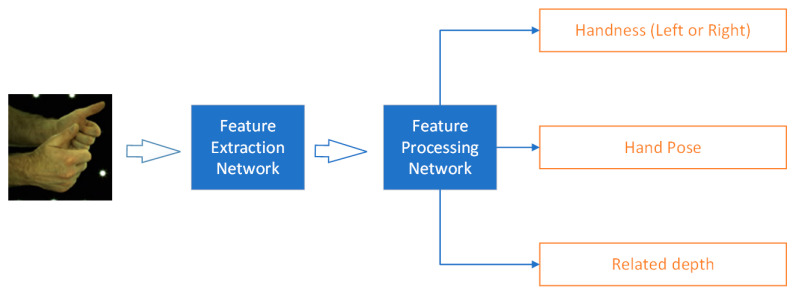
The basic operating principle of the original InterNet. For the outputs, the “handness” means the existence possibility of right hand and left hand, as the output of the fully connected layer; the “Hand Pose” means the hand pose of the right hand and left hand from transferred 2D to 3D hand key-points position; the “Related depth” means the root point related depth of two hands.

**Figure 2:**
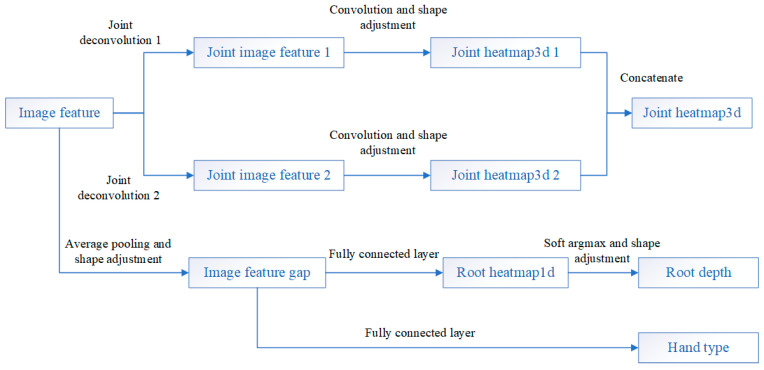
Specific structure with the input and outputs of PoseNet (feature processing network in Figure 1) is displayed. “Image feature” is the feature map obtained in the feature extraction network, “Joint heatmap3d” is the heatmap of the estimated key-points’ position of the hand in a 3D pose, “Root depth” is the root point related depth of two hands, and “Hand type” is the judged hand type (left or right).

The InterNet aims to estimate 3D hand key-points position from a single RGB image. The whole network pipeline is shown in Figure 1. It requires a set of pre-labeled RGB images for training. Take an RGB image, cut the hand area, adjust the resolution as the input, then use ResNet (with the last fully connected layer removed) as a feature extraction network to extract image features. The specific structure of the PoseNet designed in [8] is shown in Figure 2. Starting from the feature map, the PoseNet firstly used the up-sampler (noted as “Joint deconv 1&2” in Figure 2, meaning deconvolutional network) with convolutional processing and shape adjustment to build a 3D key-point heatmap for hand pose reconstruction. Additionally, PoseNet used average pooling and shape adjustment to process the feature map, then used fully connected layers to obtain hand type information (after softmax processing) and relative root key-point depth between two hands.

The author designed a combination of three loss functions, defining: (1) the binary cross-entropy function *L_h_*, which constrains the network to infer the attribution and quantity of the hands appearing in the image; (2) the L2 loss function *L_hand_*, which is used to constrain the 2.5D hand posture and estimate the accuracy of the position of the joint points; (3) L1 loss function *L_rel_*, which constrains the estimation of the Euclidean distance between the root point of the right and left hands. The three loss functions are additively combined with the same weight, and they work together for end-to-end model training.

### 3.2. Data Annotation

To annotate the key-points of the hand, Moon et al. [8] directly extended the commonly used the 21 key-points annotation method for one [11] to two hands and marked the fingertips of each finger with three joint rotation points and the root point in the palm of each hand. Moreover, this also provides the anchor point coordinates of the bounding box of a player’s position. At the same time, using the automatic labeling and manual combination, the ground truth of the manual and RootNet labels were obtained, respectively.

## 4. InterNet+

In this section, we will describe in detail the modifications we made to the original enhanced InterNet method with our initial motivation, including the improved and redesigned feature extraction network, feature map processing, and new network model training methods.

### 4.1. Redesigned Feature Extraction Network

In recent research, the MobileNet family shows greater efficiency and computational performance ratio in computer vision tasks with a small or medium amount of data than classic network backbone, such as ResNet [15]. To extract the local and global high-dimensional information contained in the RGB data more simply and efficiently, compared to the ResNet-50 used in the InterNet as the backbone of the feature extractor, we have integrated the latest in the current deep learning and neural network fields. The study’s results have redesigned a feature extraction network to achieve the goal of reducing the weight of the network and obtaining the highest possible accuracy. The following section will introduce the new technology we introduced in the self-designed feature extraction network.

#### 4.1.1. Inverted Residual Block

**Figure 3:**
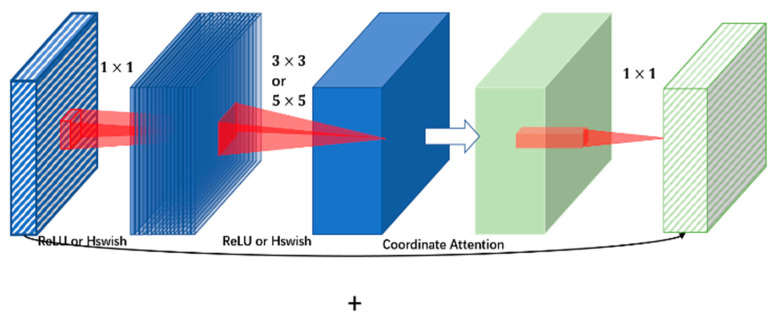
The design of our inverted residual module. Different colors represent input, hidden layer and output. Additionally, the change from blue to green represents the processing of the coordinate attention mechanism.

We refer to the design of MobileNet v2 [31] and use the inverted residual block. This module has also been applied to the series of networks in MobileNet v3 [15] and MoGA [16]. The inverted residual block module is similar to the residual block [14], consisting of an input, bottleneck, and extension. In previous studies, it has been proven that the bottleneck contains all the necessary information, and the expansion layer is only used as the implementation detail of the accompanying tensor nonlinear transformation [31]. Insert a shortcut connection similar to the residual connection directly between the bottlenecks. The difference between our block design and the residual module is shown in Figure 3. In actual applications, our design has been proven to have higher memory efficiency and experimental effects than the traditional residual modules. To ensure the light weight of the network and retain the feature information as much as possible, contrary to some networks of MobileNet v3 and MoGA, we prefer to use 3 × 3 or 5 × 5 convolutional kernels instead of 7 × 7 kernels and reduce the number of bottlenecks as much as possible.

#### 4.1.2. Coordinate Attention Mechanism

Hou et al. proposed a coordinate attention mechanism [18], which had been proven to achieve more prominent effects than the traditional spatial attention mechanisms on many tasks in the field of computer vision. Similar to the design of the MoGA network, we added a coordinate attention mechanism module instead of the SE-block (squeeze-and-excitation block) [32] before the 1 × 1 convolution at the end of the bottleneck (mainly the first bottleneck where the channels changed). The follow-up ablation analysis and control experiments demonstrate that, by adding the coordinate attention mechanism module, the hand pose estimation intensive reading can be improved, and the effect is better than the traditional spatial attention mechanism modules, such as SE-block or CBAM (convolutional block attention module) [33].

#### 4.1.3. ACON Activation Function

Ma et al. proposed the ACON activation function. They obtained motivation from the function $max\left(x_{1},x_{2},\ldots ,x_{n}\right)$ that defines the maximum value of n parameters, then determined a new smooth and differentiable approximation function, as shown in Equation (1), where β is the transformation parameter. When β approaches infinity, $S_{\beta }$ approaches maximum; when  β approaches 0, $S_{\beta }$ approaches arithmetic mean.

(1) $$S_{\beta }\left(x_{1},\ldots ,x_{n}\right)=\frac{\sum_{i=1}^{n}x_{i}e^{\beta x_{i}}}{\sum_{i=1}^{n}e^{\beta x_{i}}}$$

Among the commonly used activation functions, the structure is basically as $max\left(\eta_{a}\left(x\right),\eta_{b}\left(x\right)\right)$, where $\eta_{a,b}\left(x\right)$ represents a linear function, such as the ReLU function, max(x,0).

The author constructed the approximate fitting smooth differentiable function $S_{\beta }\left(\eta_{a}\left(x\right),\eta_{b}\left(x\right)\right)$ of $max\left(\eta_{a}\left(x\right),\eta_{b}\left(x\right)\right)$, then simplified it and obtained Equation (2).

(2) $$S_{\beta }\left(\eta_{a}\left(x\right),\eta_{b}\left(x\right)\right)=\left(\eta_{a}\left(x\right)-\eta_{b}\left(x\right)\right)\cdot \sigma \left[\beta \left(\eta_{a}\left(x\right)-\eta_{b}\left(x\right)\right)\right]+\eta_{b}\left(x\right)$$

where σ means the sigmoid function. Substituting $\eta_{a}\left(x\right)=x$ and $\eta_{b}\left(x\right)=0$, the approximate fitting of the ReLU function can be obtained as $S_{\beta }\left(x,0\right)=x\cdot \sigma \left(\beta x\right)$, which is the same as the swish function [17]. The author defined it as the ACON-A function.

Based on the above operations, the author believed that other maximum-based activation functions in the ReLU family can be transformed into the ACON family. The author used the same method to deal with the PReLU function (f(x)=max(x,0)+p·min(x,0), where p is a learnable parameter with an initial value of 0.25), and rewrite it as f(x)=max(x,px)(p<1), and get the approximate fitting, named ACON-B, as shown in Equation (3).

(3) $$f_{ACON-B}\left(x\right)=S_{\beta }\left(x,px\right)=\left(1-p\right)x\cdot \sigma \left[\beta \left(1-p\right)x\right]+px\;\;$$

Similarly, the author gives a more general function form based on the form of ACON-B function, named ACON-C, as shown in Equation (4), where $p_{1}$ and $p_{2}$ are the learnable parameters.

(4) $$f_{ACON-C}\left(x\right)=S_{\beta }\left(p_{1}x,p_{2}x\right)=\left(p_{1}-p_{2}\right)x\cdot \sigma \left[\beta \left(p_{1}-p_{2}\right)x\right]+p_{2}x$$

This activation function learns the linearity and non-linearity gate of the entire activation function area of a specific network location by setting hyperparameters to switch between active and inactive states. In this study, we use the ACON-C function. We added this activation function to the end of the separable convolution before the inverted residual block of the feature extractor and achieved good results.

**Figure 4:**
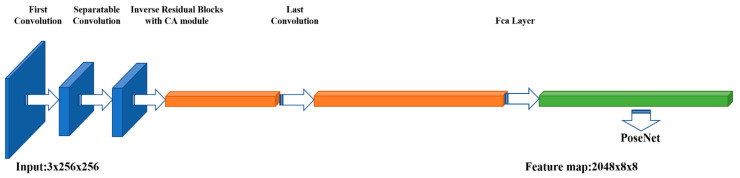
Feature extractor part of the network. Each cube represents the output tensor of a certain layer; the left and right thickness represent the number of channels, and the other two dimensions are its 2D dimensions. Orange and green represent the tensor processed by the coordinate and multispectral attention mechanisms, respectively.

Considering the latest achievements in the above deep learning field, we designed a feature extraction network with superior performance using a combination of architectures similar to MobileNet v3 and MoGA networks (without global pooling and classifiers). The structure is shown in Figure 4. See Table 1 for specific structure.

### 4.2. Processing of the Feature Maps

The hand pose estimation tasks commonly face a problem of insufficient information, whether from the local pixel aspect or from the global spectrum aspect [2,3]. To solve this problem and make better use of local and global information from single RGB image, we have introduced the latest frequency-domain attention mechanism into the network structure.

Upon obtaining the feature map through the feature extraction network, we did not directly send it to the subsequent fully connected layer and linear structure to determine whether there are left and right hands, the number of hands in the image, or to fit the location heatmap of the key-points. The feature map is a three-dimensional tensor with a number of channels much larger than the size of length and height. In our network, its size is 2048 × 8 × 8; therefore, we use a new attention mechanism to process it to preserve as much information in the frequency domain as possible before the subsequent processing.

We process the feature maps obtained from the feature extraction network through the FcaNet layer (also known as the multi-spectral attention mechanism) proposed by Qin et al. [20]. The author redefines global average pooling (GAP [34], which is often used in attention mechanisms, mainly to extract global feature representations while reducing network parameters) from a frequency domain perspective to compensate for the lack of feature information in the existing channel attention methods, and GAP is extended to a more general 2D discrete cosine transform (DCT) form, which makes full use of the information by introducing more frequent components. Processing this module, the frequency domain information of the feature map obtained in the previous period can be better retained, and the attention characteristic can be used to improve the training accuracy.

In previous hand pose estimation tasks, attention mechanism modules were rarely used. On the one hand, studies on the attention mechanism are still progressing. Many current attention mechanisms in spatial or frequency domain could not improve the performance of the network in hand pose estimation tasks. On the other hand, the attention mechanism can play a better role in promoting network performance in some datasets; nevertheless, the effect is not obvious in the other datasets and may even have the opposite effect. Considering the analysis of the principle and the continuous attempts of its correct application to the network structure, we can add multiple types of attention mechanism modules to the whole network to improve its performance on different datasets. The ablation analysis in subsequent experiments can prove the effectiveness of the functional modules in our method.

### 4.3. Effective Way of Network Training

Compared to the original InterNet, using the widely used Adam [35] as the optimizer with a simpler network learning method that decreases the learning rate after specific epochs, we use a better AdamW [36] optimizer and cosine annealing learning rate (cosine annealing and warm restart) [37]. This learning method makes the learning rate self-decay within the time period multiplied by the regularity. Therefore, a better performance model can be trained at a specific epoch, making the training of the network more controllable.

## 5. Experiment

We evaluated our InterNet+ network on RGB datasets used for hand pose estimation, including the STB and RHD datasets, and the feasibility test conducted on the incomplete InterHand2.6M dataset (5 fps version) [8]. We compared the original InterNet network to other similar network methods and used diagrams to illustrate the advantages of our method. Thereafter, we performed ablation analysis, and the distribution proved the effectiveness of the functional modules used in our method.

### 5.1. Datasets

We conducted experiments mainly on the STB and RHD datasets based on the proposed InterNet+ network, with both of them having achieved considerable accuracy improvements compared to the original model, and achieved state-of-the-art results. At the same time, in order to prove the effectiveness of the method, we also conducted a small number of experiments on InterHand2.6M dataset (5 fps version). Referring to reference [2] and our previous review research [21], we here briefly introduce the datasets and related conditions of the experiment.

#### 5.1.1. STB Dataset

The stereo hand pose tracking benchmark (STB) [38] is one of the most widely used hand pose estimation datasets. The author simultaneously captured both stereo and depth images from a Point Grey Bumblebee2 stereo imaging device with Intel Real Sense F200 active depth cameras. It contains a total of 12 action sequences performed by one person in six different backgrounds. Each sequence contains 1500 frames, for a total of 18,000 frames (15,000 for training and 3000 for evaluation). The resolution is 640 × 480, and the annotation contains the depth map and 3D position information of the 21 key-points.

#### 5.1.2. RHD Dataset

The Rendered Handpose Dataset (RHD) [11] is also one of the most widely used hand pose estimation datasets. The author considered that the previous datasets have problems such as limited changes, insufficient number of available samples, and incomplete labeling. Therefore, a new dataset is proposed for network training. The author used the freely available three-dimensional human body model and the corresponding animation in the website Mixamo, and then used the opensource software Blender to render the images with random background.

The dataset uses 20 characters to perform 39 actions, totaling 43,986 frames (41,258 for training and 2728 for evaluation). The resolution is 320 × 320, providing depth segmentation mask and 2D and 3D position annotation information of 21 key-points.

#### 5.1.3. InterHand2.6M Dataset (5fps/30fps)

Compensating for the insufficiencies in the previous datasets for the identification of interactive gestures and multi-hand targets, [12] proposed this dataset. The data is captured in a multi-camera studio consisting of 80–140 cameras capturing at 30–90 frames-per second (fps). The dataset has a total of 2,590,347 frames (1,361,062, 380,125, and 849,160 frames in the training, verification, and test sets, respectively), and the resolution is 512 × 334. It also contains annotations generated by RootNet and manual annotations. The incomplete version of the 5 fps is currently open (a total of 1,275,786 frames).

### 5.2. Experimental Environment and Results

Our network model is trained in the following environment:

System: ubuntu 16.04 with 64G RAM

CPU: Intel Xeon E5-2620 × 32 GPU: NVIDIA GeForce RTX 2080Ti × 2.

We use widely used mean end point error (EPE, according to reference [3], which is defined as a mean Euclidean distance between the predicted and ground-truth 3D hand pose after root joint alignment) as the evaluation metrics for STB dataset and RHD dataset. The definition of the mean EPE is as follows, we have our prediction hand key-points coordinates $H_{pre}$ and the ground-truth of the hand key-points coordinates $H_{gt}$. Then, make root joint alignment operation, translate all the coordinates of $H_{pre}$ as $H_{pre}^{\prime }$ to get the root key-point the same as the root point in $H_{gt}$, and we obtain EPE as Equation (5), where *N* represents the number of the hand key-points in single image.

(5) $$\mathrm{EPE}=\frac{1}{N}\underset{N}{\sum }{∥H_{pre}^{\prime }-H_{gt}∥}_{2}$$

The training method adopts the cosine annealing learning rate method with an initial period of two and a magnification of three. Considering experience, the best result is generally obtained near the epoch where the learning rate is about to converge to 0; hence, the STB result is taken from 45 epochs (if the convergence is fast enough, sometimes the best result will appear at 44 epochs), and the RHD training result is taken from 189 epochs.

**Figure 5:**
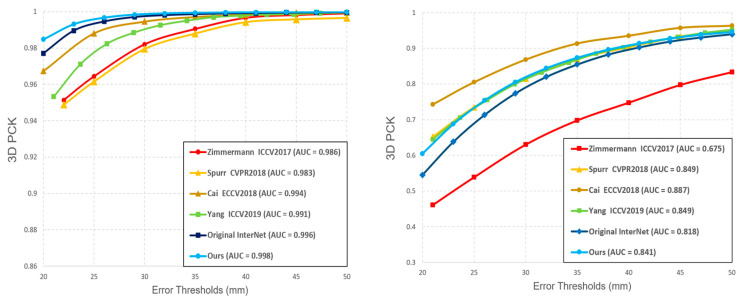
Quantitative evaluation for different methods on STB dataset and RHD dataset. (**Left**): 3D PCK on STB dataset. (**Right**): 3D PCK on RHD dataset.

**Figure 6:**
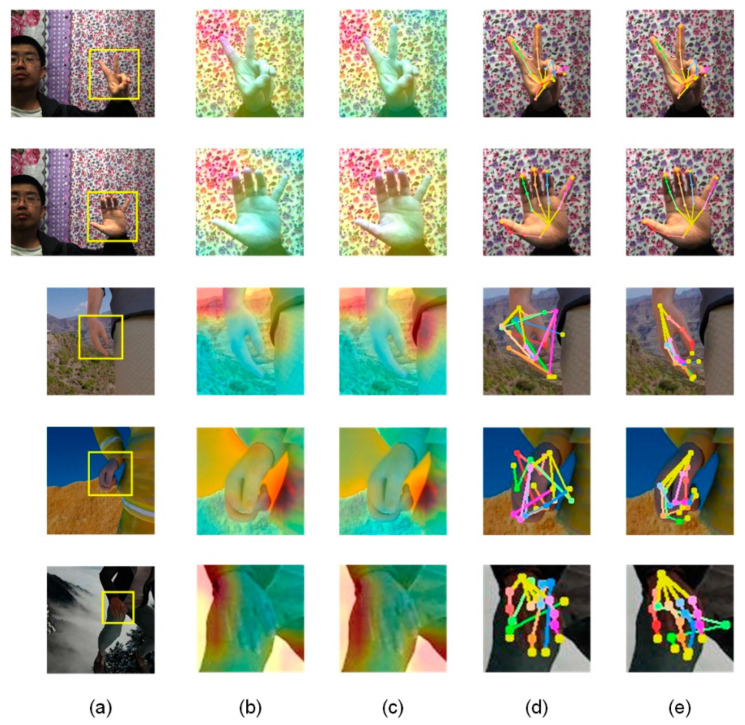
Examples comparing to the feature maps and outputs of the original InterNet with ours. The first two rows and the last three rows are examples from the STB dataset and RHD dataset. Columns (**a**) are the original images from datasets, where the yellow box means the hand area separated by bounding box, Columns (**b**,**d**) are from the original net, and columns (**c**,**e**) are ours. In feature maps like column (**b**,**c**), the background features are easier to be identified by global feature extraction, consequently have more attention. The accurate recognition of background features helps the fol-lowed up PoseNet to process hand pose estimation.

The comparison of our hand pose estimation results with the original InterNet and other methods is shown in Table 2, and the statistic comparison of PCK (percentage of correct key-points) and AUC (the area under the curve) is shown in Figure 5. Additionally, the demonstration of feature maps (as the outputs of the feature extraction network) with the final outputs between the original InterNet and those of our network is shown in Figure 6. From what we can see, our model has a more accurate estimation in simple background occasions and better performance than the original network in complex backgrounds in which hands are difficult to be distinguished.

The comparison and analysis reveal that our method has a greater improvement in the accuracy of hand pose estimation compared to other similar methods. Moreover, compared to the original InterNet, our network has a smaller data volume, lighter weight, and faster speed under the same conditions in the experiment as shown in Table 3. Additionally, the inference time comparison between the original network and ours is shown in Table 4. From this, we can see that, although our model has better performance in data volume and training speed, the performance is slightly reduced in real-time learning (when batch size as 1). However, as the batch size increases, the performance decline is offset, and a better performance is obtained.

**Figure 7:**
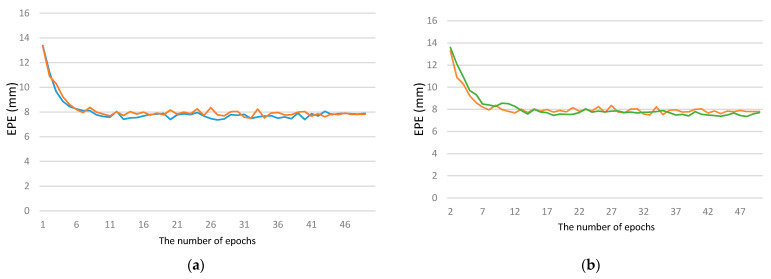
Convergence comparison of InterNet+ and InterNet on the STB dataset. The blue and orange lines in (**a**) are the two complete trainings process of the original InterNet under the same conditions. It can be observed that the training effect has a large randomness in the early stage and stabilizes after 45 epochs. The green line in (**b**) is the training method we used, and it can be seen that the overall convergence fluctuates regularly with the change in the learning rate. After 25 epochs, it is basically stabilized.

To better demonstrate the convergence of our method, we compared the convergence process that achieved the best results on the STB dataset within 50 epochs to the original method, and the results are shown in Figure 7.

Considering the InterHand2.6M dataset, we use mean per joint position error (MPJPE, which is defined as a Euclidean distance between predicted and ground-truth 3D joint locations after root joint alignment [3], a definition similar to that of EPE, but here we keep the same with the original InterNet) to define the standard for hand pose estimation, and the comparison with the original InterNet experimental results is shown in Table 5.

This experiment verifies the usability of our method on multi-target hands and interactive gestures and its ability to equivalently replace the original network. The comparative analysis also demonstrates that our method can still achieve better results in accurate single-handed target recognition than the original method using less data.

### 5.3. Ablation Analysis

Regarding the design and layout of the functional modules we used in the network, we conducted ablation analysis experiments on the STB and RHD datasets to verify the effectiveness of the functional modules we used.

#### 5.3.1. Coordinate Attention Mechanism Module

Compared to the background, the hand target often occupies a smaller proportion of the pixels, and there are many problems, such as insufficient resolution and incomplete information. The introduction of the attention mechanism can theoretically promote the network’s focused cognition and feature extraction of the target area.

We evaluated the feasibility of introducing the newly proposed coordinate attention mechanism in the feature extraction process of the hand pose estimation network. We carried out experimental comparisons on the STB and RHD datasets, respectively. Thereafter, we verified the effectiveness of the coordinate attention mechanism module in the network of the feature extractor. We compared it to the previous SE attention mechanism module, and we observed that the coordinate attention mechanism is better for improving network performance. The experimental results are shown in Table 6.

#### 5.3.2. Processing of Feature Map by Using the FcaNet Layer

In the previous study, the attention mechanism module was seldom used in the tasks of hand pose estimation. We perceive such vacancies in network design. Owing to the particularity of the hand pose estimation task, we introduced a new attention mechanism into the network to improve the performance of the network. Based on the better results of the module based on the coordinate attention mechanism, we performed preliminary processing on the extracted feature maps to facilitate the subsequent operations.

We also evaluated the feasibility of using a new frequency-domain attention mechanism in the processing of the feature map of the hand pose estimation network. Similarly, we conducted experiments on the STB and RHD datasets, respectively. This verified the effectiveness of the multi-spectral attention mechanism module in the hand pose estimation network feature extractor. The experimental results are shown in Table 7.

## 6. Discussion and Outlook

Since the publication of the InterNet network and the InterHand2.6M dataset, studies on the interactive and multi-objective hand pose estimation have continued to advance. Based on the original infrastructure of the InterNet, we redesigned its feature extraction part to make a better use of the input information to achieve a greater improvement in performance. Considering the continuous development in the field of deep learning as well as computer vision, we can expect the following developments to positively impact studies in the field:

- The development of feature extraction backbone. Recently, scholars have re-examined the widely used convolutional neural network backbones such as ResNet, made new improvements, and targeted adjustments to training strategies [42]. These improvements will help the development of many tasks in the field of computer vision, including hand pose estimation;
- The rise of Transformer in the field of computer vision [43]. Previously, Transformer was mostly used in fields such as natural language processing [44]. Since its introduction into the field of computer vision, it has demonstrated amazing capabilities in a variety of visual tasks, such as segmentation and classification. At present, visual Transformer has been introduced into the field of attitude estimation [45]. Therefore, the modified visual Transformer may bring greater changes in the field of hand posture estimation and overall changes in the network architecture;
- Considering the data acquisition for hand posture estimation, scholars are gradually replacing manual labeling with automatic or semi-automatic methods. Using neural networks and other learning models for more accurate labeling can help reduce the workload caused by manual labeling.

Still, there are some limitations in our research. To begin with, due to the need to ensure the hand pose estimation function of the whole network, we did not make innovative changes to the feature processing network, “PoseNet”. Secondly, due to equipment reasons, we have not yet been able to make inferences on the full version of the InterHand2.6M dataset. In addition, we have not yet verified the versatility of our feature extraction network for other human pose estimation tasks.

In our future research, we will verify our network’s effectiveness and accuracy performance on the full version of InterHand2.6M dataset. Furthermore, we will start with the loss function to reduce the parameters amount in the whole network. In the current thinking, we plan to use a supervised loss to define different hand joints in different scenarios to calculate the total geometric deviation. To appropriately exclude unnecessary hand joints from the loss calculation to reduce the amount of calculation.

From the other aspect, on the basis of improving network effectiveness performance and accuracy, we will try to improve functionality, such as the interaction scenarios between hands and objects. In addition, we will apply our feature extraction network to other human pose estimation tasks to verify versatility.

## Figures and Tables

**Table 1:** The structure of feature extraction network in InterNet+.

id	Name	Kernel	Attention	Activation	Dimensionality
-	Input	-	-	-	3 × 256 × 256
1	First Conv	3 × 3	-	Hswish	16 × 128 × 128
2	Separable Conv 1	3 × 3	-	ReLU	16 × 128 × 128
3	Separable Conv 2	1 × 1	-	MetaAconC	16 × 128 × 128
4	Inverted Residual 1	5 × 5	CA ^1^	ReLU	24 × 64 × 64
5	Inverted Residual 2	3 × 3	-	ReLU	24 × 64 × 64
6	Inverted Residual 3	5 × 5	CA	ReLU	40 × 32 × 32
7	Inverted Residual 4	3 × 3	-	ReLU	40 × 32 × 32
8	Inverted Residual 5	5 × 5	-	ReLU	40 × 32 × 32
9	Inverted Residual 6	5 × 5	CA	Hswish	80 × 16 × 16
10	Inverted Residual 7	5 × 5	-	Hswish	80 × 16 × 16
11	Inverted Residual 8	5 × 5	-	Hswish	80 × 16 × 16
12	Inverted Residual 9	5 × 5	CA	Hswish	128 × 16 × 16
13	Inverted Residual 10	3 × 3	-	Hswish	128 × 16 × 16
14	Inverted Residual 11	3 × 3	CA	Hswish	240 × 16 × 16
15	Inverted Residual 12	3 × 3	CA	Hswish	480 × 16 × 16
16	Inverted Residual 13	3 × 3	CA	Hswish	960 × 16 × 16
17	Last Conv	1 × 1	-	Hswish	1280 × 8 × 8
18	FcaNet Layer	-	FcaNet ^2^	-	1280 × 8 × 8
19	Final Conv	1 × 1	-	LeakyReLU	1280 × 8 × 8

^1^ CA represents the coordinate attention mechanism module mentioned above. ^2^ FcaNet represents the multi-spectral attention mechanism module.

**Table 2:** Comparison of various methods on the accuracy of hand pose estimation on the STB and RHD datasets.

Methods	GT S ^2^	GT H	EPE (STB)	EPE (RHD)
Zimmermann. et al. [11]	√ ^1^	√	8.68	30.42
Yang et.al. [39]	√	√	8.66	19.95
Chen et.al. [40]	√	√	10.95	24.20
Spurr et.al. [41]	√	√	8.56	19.73
Spurr et.al. [41]	×	×	9.49	22.53
InterNet [8]	×	×	7.95	20.89
**InterNet+ (ours)**	×	×	**7.38**	**19.30**

^1^ The selected mark indicates the method of using ground truth in the inference time. ^2^ S and H represent the scale and handness, respectively.

**Table 3:** The comparison of the parameters of our method and the original InterNet and the speed of the experiment.

	Total Parameters (M)	Parameters Size (MB)	Time Per Iteration (s) ^1^
InterNet	23.51	89.68	0.61
InterNet+ (ours)	**11.12**	**42.42**	**0.58**

^1^ represents the situation of “cudnn.benchmark = True”.

**Table 4:** The inference performance (frames processed per second) comparison of original model and ours in RHD dataset.

Batch Size	1	4	8	16	32
InterNet	32.46	136.35	194.79	247.52	257.26
InterNet+ (ours)	28.41	101.32	151.50	227.36	296.41

**Table 5:** Comparison of MPJPE (mean per joint position error) between our results and that of the original InterNet on the InterHand2.6M dataset.

	Single Hand	Interacting Hands
InterNet	12.16	16.02
InterNet+ (ours)	**11.67 ^1^**	17.63 ^1^

**^1^** Represents the result of the network training on the 5fps incomplete version dataset.

**Table 6:** The ablation experiment for coordinate attention mechanism module (the number of training epochs in the ablation experiment is consistent with the training state of the complete network); the evaluation is measured by EPE (mm).

Model	STB	RHD
InterNet+ (without CA module)	7.44	19.36
InterNet+ (with SE module)	7.76	19.45
InterNet+ (ours)	**7.38**	**19.30**

**Table 7:** Ablation experiment of the FcaNet module on different datasets (the number of training epochs in the ablation experiment is consistent with the training state of the complete network), the evaluation is measured by EPE (mm).

Net Architecture	STB	RHD
InterNet+ (without FcaNet layer)	7.52	20.01
InterNet+ (ours)	**7.38**	**19.30**

## Data Availability

Data available in a publicly accessible repository. Data citation: [dataset] Zhang, J.; Jiao, J.; Chen, M.; Qu, L.; Xu, X.; Yang, Q. 2017. The stereo hand pose tracking benchmark (STB); https://github.com/zhjwustc/icip17_stereo_hand_pose_dataset; Version (if any); DOI: 10.1109/ICIP.2017.8296428; [dataset] Zimmermann, C.; Brox, T. 2017. The Rendered Handpose Dataset (RHD); https://github.com/lmb-freiburg/hand3d; Version 1.1; DOI: 10.1109/ICCV.2017.525; [dataset] Moon, G.; Yu, S.-I.; Wen, H.; Shiratori, T.; Lee, K.M. 2020. InterHand2.6M; https://mks0601.github.io/InterHand2.6M; Version 0.5; DOI: 10.1007/978-3-030-58565-5_33.

## References

[1] Zhao S., Zhang L., Shen Y., Zhao S., Zhang H. (2019). Super-resolution for monocular depth estimation with multi-scale sub-pixel convolutions and a smoothness constraint. IEEE Access.

[2] Chatzis T., Stergioulas A., Konstantinidis D., Dimitropoulos K., Daras P. (2020). A comprehensive study on deep learning-based 3D hand pose estimation methods. Appl. Sci..

[3] Doosti B. (2019). Hand Pose Estimation: A Survey. arXiv.

[4] Oberweger M., Lepetit V. DeepPrior++: Improving Fast and Accurate 3D Hand Pose Estimation. Proceedings of the 2017 IEEE International Conference on Computer Vision Workshops (ICCVW).

[5] Zhang Z., Xie S., Chen M., Zhu H. (2001). Hand Augment: A Simple Data Augmentation Method for Depth-Based 3D Hand Pose Estimation. arXiv.

[6] Tompson J., Stein M., Lecun Y., Perlin K. (2014). Real-time continuous pose recovery of human hands using convolutional networks. ACM Trans. Graph..

[7] Rong Z., Kong D., Wang S., Yin B. RGB-D Hand Pose Estimation Using Fourier Descriptor. Proceedings of the 2018 7th International Conference on Digital Home (ICDH).

[8] Moon G., Yu S.-I., Wen H., Shiratori T., Lee K.M. (2020). InterHand2.6M: A dataset and baseline for 3D interacting hand pose estimation from a single RGB image. Computer Vision–ECCV 2020 (Lecture Notes in Computer Science).

[9] Ge L., Ren Z., Li Y., Xue Z., Wang Y., Cai J., Yuan J. 3D Hand Shape and Pose Estimation from a Single RGB Image. Proceedings of the 2019 IEEE/CVF Conference on Computer Vision and Pattern Recognition (CVPR).

[10] Yang L., Li S., Lee D., Yao A. Aligning Latent Spaces for 3D Hand Pose Estimation. Proceedings of the 2019 IEEE/CVF International Conference on Computer Vision (ICCV).

[11] Zimmermann C., Brox T. Learning to Estimate 3D Hand Pose from Single RGB Images. Proceedings of the 2017 IEEE International Conference on Computer Vision (ICCV).

[12] Zhang J., Jiao J., Chen M., Qu L., Xu X., Yang Q. (2016). 3D Hand Pose Tracking and Estimation Using Stereo Matching. arXiv.

[13] Ge L., Liang H., Yuan J., Thalmann D. (2018). Robust 3D hand pose estimation from single depth images using multi-view CNNs. IEEE Trans. Image Process..

[14] Hy K., Zhang X., Ren S., Sun J. Deep Residual Learning for Image Recognition. Proceedings of the 2016 IEEE Conference on Computer Vision and Pattern Recognition (CVPR).

[15] Howard A., Sandler M., Chu G., Chen L.-C., Chen B., Tan M., Wang W., Zhu Y., Pan R., Vasudevan V. (2019). Searching for MobileNetV3. arXiv.

[16] Chu X., Zhang B., Xu R. (2019). MoGA: Searching beyond MobileNetV3. arXiv.

[17] Ramachandran P., Zoph B., Le Q.V. (2017). Searching for Activation Functions. arXiv.

[18] Hou Q., Zhou D., Feng J. (2021). Coordinate Attention for Efficient Mobile Network Design. arXiv.

[19] Ma N., Zhang X., Sun J. (2020). Activate or Not: Learning Customized Activation. arXiv.

[20] Qin Z., Zhang P., Wu F., Li X. (2020). FcaNet: Frequency Channel Attention Networks. arXiv.

[21] Liu Y., Jiang J., Sun J. Hand Pose Estimation from RGB Images Based on Deep Learning: A Survey. Proceedings of the 2021 IEEE 7th International Conference on Virtual Reality (ICVR).

[22] Oberweger M., Wohlhart P., Lepetit V. (2015). Hands Deep in Deep Learning for Hand Pose Estimation. arXiv.

[23] Chang J.Y., Moon G., Lee K.M. V2V-PoseNet: Voxel-to-Voxel Prediction Network for Accurate 3D Hand and Human Pose Estimation from a Single Depth Map. Proceedings of the 2018 IEEE/CVF Conference on Computer Vision and Pattern Recognition.

[24] Zhu T., Sun Y., Ma X., Lin X. Hand Pose Ensemble Learning Based on Grouping Features of Hand Point Sets. Proceedings of the 2019 IEEE/CVF International Conference on Computer Vision Workshop (ICCVW).

[25] Cai Y., Ge L., Cai J., Yuan J. (2018). Weakly-supervised 3D hand pose estimation from monocular RGB images. Computer Vision–ECCV 2018 (Lecture Notes in Computer Science).

[26] Li M., Gao Y., Sang N. (2020). Exploiting Learnable Joint Groups for Hand Pose Estimation. arXiv.

[27] Chen X., Liu X., Ma C., Chang J., Wang H., Chen T., Guo X., Wan P., Zheng W. (2021). Camera-Space Hand Mesh Recovery via Semantic Aggregation and Adaptive 2D-1D Registration. arXiv.

[28] Chen Y., Tu Z., Kang D., Bao L., Zhang Y., Zhe X., Chen R., Yuan J. (2021). Model-based 3D Hand Reconstruction via SELF-Supervised Learning. arXiv.

[29] Doosti B., Naha S., Mirbagheri M., Crandall D.J. HOPE-Net: A Graph-Based Model for Hand-Object Pose Estimation. Proceedings of the 2020 IEEE/CVF Conference on Computer Vision and Pattern Recognition (CVPR).

[30] Chen Y., Tu Z., Kang D., Chen R., Bao L., Zhang Z. (2021). Joint hand-object 3D reconstruction from a single image with cross-branch feature fusion. IEEE Trans. Image Process..

[31] Sandler M., Howard A., Zhu M., Zhmoginov A., Chen L.-C. (2018). MobileNetV2: Inverted Residuals and Linear Bottlenecks. arXiv.

[32] Hu J., Shen L., Albanie S., Sun G., Wu E. (2017). Squeeze-and-Excitation Networks. arXiv.

[33] Woo S., Park J., Lee J.-Y., Kweon I.S. (2018). CBAM: Convolutional Block Attention Module. arXiv.

[34] Lin M., Chen Q., Yan S. (2013). Network in Network. arXiv.

[35] Kingma D.P., Ba J. (2014). Adam: A method for Stochastic Optimization. arXiv.

[36] Li D., Blake C.H., Nidever D., Halverson S.P. (2018). Temporal Variations of Telluric Water Vapor Absorption at Apache Point Observatory.

[37] Loshchilov I., Hutter F. (2017). Decoupled Weight Decay Regularization. arXiv.

[38] Zhang J., Jiao J., Chen M., Qu L., Xu X., Yang Q. A Hand Pose Tracking Benchmark from Stereo Matching. Proceedings of the 2017 IEEE International Conference on Image Processing (ICIP).

[39] Yang L., Yao A. Disentangling Latent Hands for Image Synthesis and Pose Estimation. Proceedings of the 2019 IEEE/CVF Conference on Computer Vision and Pattern Recognition (CVPR).

[40] Chen L., Lin S.-Y., Xie Y., Tang H., Xue Y., Xie X., Lin Y.-Y., Fan W. (2018). Generating Realistic Training Images Based on Tonality-Alignment Generative Adversarial Networks for Hand Pose Estimation. arXiv.

[41] Spurr A., Song J., Park S., Hilliges O. Cross-Modal Deep Variational Hand Pose Estimation. Proceedings of the 2018 IEEE/CVF Conference on Computer Vision and Pattern Recognition.

[42] Bello I., Fedus W., Du X., Cubuk E.D., Srinivas A., Lin T.-Y., Shlens J., Zoph B. (2021). Revisiting ResNets: Improved Training and Scaling Strategies. arXiv.

[43] Han K., Wang Y., Chen H., Chen X., Guo J., Liu Z., Tang Y., An X., Cu C., Xu Y. (2020). A Survey on Visual Transformer. arXiv.

[44] Devlin J., Chang M.-W., Lee K., Toutanova K. (2018). BERT: Pre-Training of Deep Bidirectional Transformers for Language Understanding. arXiv.

[45] Zheng C., Zhu S., Mendieta M., Yang T., Chen C., Ding Z. (2021). 3D Human Pose Estimation with Spatial and Temporal Transformers. arXiv.

